# Inflammatory cytokines and quality of life response to weight reduction in obese patients with bronchial asthma

**DOI:** 10.4314/ahs.v25i1.33

**Published:** 2025-03

**Authors:** Shehab M Abd E-Kader, Neveen Refaey, Afnan M AlKhateeb, Moataz Al-Madaah, Saad S AlFawaz, Ziyad A Neamatallah, Umar M Alabasi, Hajed Alotaibi, Mazen Homoud, Riziq Allah Mustafa Gaowgzeh, Salwa R El-Gendy, Mohamed F El-Banna, Heba Embaby, Fatma A Hegazy, Rasha M Hegazy, Nahla Khalefa, Khaled M Mounir, Saif Mehmed, Mohamed Y Abdelsamee, Ahmed M Aboeleneen

**Affiliations:** 1 Department of Physical Therapy, Faculty of Medical Rehabilitation Sciences, King Abdulaziz University, Jeddah, Saudi Arabia; 2 Department of Cardiopulmonary and Geriatrics, Faculty of Physical Therapy, Cairo University, Egypt; 3 Department of Women Health, Faculty of Physical Therapy, Cairo University, Egypt; 4 Department of Respiratory Therapy, Faculty of Medical Rehabilitation Sciences, King Abdulaziz University, Jeddah, Saudi Arabia; 5 Department of Pediatrics, Faculty of Physical Therapy, Cairo University, Egypt; 6 Department of Physiotherapy, College of Health Sciences, University of Sharjah, United Arab Emirate; 7 Department of Neurology, Faculty of Physical Therapy, Cairo University, Egypt; 8 Faculty of Physical Therapy, Galala University, Egypt; 9 Department of Basic Sciences, Faculty of Physical Therapy, Suez University, Egypt; 10 Department of Basic Sciences, Faculty of Physical Therapy, Cairo University, Egypt

**Keywords:** Bronchial Asthma, Cytokines, Obesity, Quality of Life, Weight Reduction

## Abstract

**Background:**

Globally, about 20% of the population are affected with asthma. However, public health is adversely affected with asthma and obesity.

**Objective:**

The target of the present study was to measure influence of weight loss on quality of life and Inflammatory cytokines of obese asthmatic patients.

**Material and Methods:**

Eighty obese asthmatic patients; the mean of their age was 42.71 ± 6.35 year and body mass index (BMI) was 32.85 ± 3.16 Kg/m2. Participants equally assigned in group (A) received weight reducing program, where group (B) received no therapeutic intervention.

**Results:**

The Health-related quality of life (SF-36 HRQL) subscale scores, interleukin-10 (IL-10) and Asthma Control Test (ACT) improved significantly, where, the mean values of BMI, TNF-α and IL-6 were significantly reduced in group (A). While group (B) had no significant changes in their parameters. In addition, the differences between both groups were not significant at the end of the study.

**Conclusion:**

Inflammatory cytokines and quality of life parameters improved with lifestyle modification among obese asthmatic patients.

## Introduction

Asthma is a respiratory disorder characterized by chronic airway inflammation of airways that currently affect 300 million populations and expected to affect about 400 million people worldwide by 2025^1^,^2^. However, obesity affects about 500 million subjects globally^3^. One of the cardinal features of asthma is systemic inflammation^4^,^5^, that worsen the parameters of lung function and patient's general condition^6^–^8^ through airway inflammation and increased frequency of exacerbations^9^.

Excessive body weight increases the risk for asthma as about 40-90% of obese subjects develop asthma^10^. Moreover, obesity increases the severity of respiratory symptoms of asthma^11^,^12^, in addition to make it difficult to control asthma as it negatively influences parameters of lung function^13^-^15^. As a result, obese asthmatic patients had poor quality of life^16^.

Limited studies that involve weight reducing program for asthma patients. In one previous study, weight loss was associated with improved lung function parameters, control asthma symptoms and overall quality of life among obese asthmatic subjects^17^. However, bariatric surgery resulted in good asthma control and lung function^18^,^19^. Therefore, the target of the present study was to measure influence of weight loss on quality of life and Inflammatory cytokines of obese asthmatic patients.

## Subjects and Methods

Eighty obese patients with asthma of both sex; their age mean was 42.71 ± 6.35 year and their body mass index (BMI) mean was 32.85 ± 3.16 Kg/m^2^. Participants with cardiovascular, hepatic and renal disorders were excluded. The selected participants were assigned into two groups. Group (A) received weight reducing program consisted of treadmill walking exercise and diet control, while group (B) was considered as a control group received no intervention. The Scientific Research Ethical Committee, Faculty of Applied Medical Sciences at King Abdulaziz University, approved this study. All participants signed the consent and were free to withdraw from the study at any time.

### Measurements

- Inflammatory cytokines measurements: Overnight fasting venous blood samples were drained and centrifuged to measure Interleukin-6 (IL-6), IL-2, IL-8, IL-4, C-reactive protein (CRP) levels were analyzed by “Immulite 2000”. However, ELISA kits (ELX 50) measured TNF-α levels.
- Quality of life (SF-36): The SF-36 is a questionnaire for detecting the quality of life and the general health changes over the last year^21^.
- Asthma Control Assessment: It is a reliable measure to detect level of asthma control over time [26] by answering 5 questions related to asthma, each question to be answered on a scale of five points with the total score of the measure equal 25 and if total score ≤ 19 referee to poor control of asthma^22^-^25^.

Clinical evaluations and laboratory analysis were performed by independent assessors who were blinded to group assignment and not involved in the routine treatment of the patients

### Procedures

1. Group (A): Participants received aerobic treadmill exercise training for 12 weeks according to the standard recommendation of exercise training. Training included warm up for 5 minutes, thirty minutes of 60-70% of maximum heart rate aerobic exercise training that followed by 10 minutes cooling down. Participants had three training sessions weekly for three months. In addition, a dietician supervised diet regimen, which provides 1200 Kilocalories/day for 12 weeks.
2. Group (B): Participants of group (B) received no clinical intervention.

## Results

The two groups were homogeneous as there were no significant differences between both groups in the baseline criteria (Table 1).

**Table 1:** baseline criteria of all participants

Parameters	Group (A)	Group (B)
**Age** (year)	43.26 ± 6.98	42.72 ± 7.53
**Gender** (F/M)	24/16	22/18
**Disease duration(**year)	11.35 ±2.17	10.61 ± 2.42
**Hemoglobin** (gm/dl)	11.88 ± 2.42	12.12 ± 2.39
**FVC** (L)	2.97 ± 1.36	3.26 ± 1.43
**FEV_1_**(L)	1.88 ± 0.79	2.18 ± 0.84
**FEV_1_/FVC** (%)	62.65 ± 8.21	64.23 ± 8.18
**FEF**_25-75%_(L/s)	1.52 ± 0.68	1.61 ± 0.72

FVC= forced vital capacity; FEV1= forced expiratory volume in the first second; FEV1/FVC= Ratio between forced expiratory volume in the first second and forced vital capacity; FEF25-75= forced expiratory flow during the middle half of the FVC.

The mean values of Health-related quality of life (SF-36 HRQL) subscale scores, interleukin-10 (IL-10) and Asthma Control Test (ACT) improved significantly, where, the mean values of BMI, TNF-α and IL-6 were significantly reduced in group (A) (Table 6 and 7). While group (B) had no significant changes in their parameters (Table 4 and 5). In addition, the differences between both groups were not significant at the end of the study (Table 6 and 7).

**Table 4:** Mean value and significance of BMI, ACT, TNF-α IL-6 and IL-10 of group (B)

Parameters	Mean±SD		t- value	Significance
	Before	After		
**BMI** (kg/m^2^)	33.23 ± 4.21	33.61 ± 4.28	1.39	P>0.05
**ACT (0–25)**	19 (17–22)	18(16–20)	1.28	P>0.05
**TNF-α** (pg/mL)	6.51 ± 1.76	6.81 ± 1.75	1.14	P>0.05
**IL-6** (pg/mL)	2.85 ± 0.83	3.24 ± 0.92	1.23	P>0.05
**IL-10** (pg/ml)	6.11 ± 1.61	5.85 ± 1.68	1.35	P>0.05

BMI= Body mass index; ACT: Asthma control test; IL-6=Interleukin-6; IL-10=Interleukin-10 and TNF-α= Tumor necrotic factor-alpha.

**Table 5:** Mean value and significance of SF-36 subscale gscores in group (B) before and at the end of the study

SF-36 subscale variables	Mean ± SD		t-value	Significance
	Before	After		
**SF-36: Health transition**	2.56 ± 0.91	2.85 ± 0.97	0.96	P>0.05
**SF-36: Physical functioning**	76.89 ± 7.96	75.11 ± 7.93	1.42	P>0.05
**SF-36: Role functioning: Physical**	79.83 ± 8.25	78.96 ± 8.24	1.18	P>0.05
**SF-36: Bodily pain**	72.54 ± 7.82	75.13 ± 7.95	1.27	P>0.05
**SF-36: General health**	72.51 ± 6.62	71.45 ± 6.58	1.31	P>0.05
**SF-36: Vitality**	56.13 ± 5.91	54.62 ± 5.73	1.15	P>0.05
**SF-36: Social functioning**	88.42 ± 7.65	87.12 ± 7.66	1.24	P>0.05
**SF-36: Role functioning: Emotional**	89.26 ± 8.65	92.35 ± 8.70	1.35	P>0.05
**SF-36: Mental health**	83.55 ± 7.29	84.62 ± 7.31	1.28	P>0.05

(*) indicates a significant difference between the two groups; P < 0.05.

**Table 6:** Mean value and significance of BMI, ACT, TNF-α, IL-6 and IL-10 of group (A) and group (B) at the end of the study

Parameters	Mean ±SD		t-value	Significance
	Group (A)	Group (B)		
**BMI** (kg/m^2^)	28.12 ± 4.15*	33.61 ± 4.28	5.62	P<0.05
**ACT** (0–25)	22 (18–25)*	18 (16–20)	5.18	P<0.05
**TNF-α** (pg/mL)	4.13 ± 1.72*	6.81 ± 1.75	4.75	P<0.05
**IL-6** (pg/mL)	2.27 ± 0.85*	3.24 ± 0.92	4.12	P<0.05
**IL-10** (pg/ml)	8.23 ± 1.68*	5.85 ± 1.68	4.81	P<0.05

BMI= Body mass index; ACT: Asthma control test; IL-6 = Interleukin-6; TNF-α= Tumor necrotic factor-alpha; CRP = C- reactive protein

(*) indicates a significant difference between the two groups, P < 0.05

**Table 7:** Mean value and significance of SF-36 subscale scores in group (A) and group (B) at the end of the study

SF-36 subscale variables	Mean ± SD		t-value	Significance
	Group (A)	Group (B)		
**SF-36: Health transition**	1.91 ± 0.83*	2.85 ± 0.97	4.63	P<0.05
**SF-36: Physical functioning**	80.31 ± 8.25*	75.11 ± 7.93	6.28	P<0.05
**SF-36: Role functioning: Physical**	86.42 ± 8.36*	78.96 ± 8.24	5.61	P < 0.05
**SF-36: Bodily pain**	69.36 ± 7.23*	75.13 ± 7.95	4.52	P<0.05
**SF-36: General health**	76.65 ± 8.12*	71.45 ± 6.58	5.64	P<0.05
**SF-36: Vitality**	64.31 ± 7.26*	54.62 ± 5.73	6.17	P<0.05
**SF-36: Social functioning**	93.54 ± 8.15*	87.12 ± 7.66	5.23	P<0.05
**SF-36: Role functioning: Emotional**	85.63 ± 8.27*	92.35 ±8.70	4.11	P<0.05
**SF-36: Mental health**	79.52 ± 6.41*	84.62 ± 7.31	4.29	P<0.05

(*) indicates a significant difference between the two groups; P < 0.05.

## Discussion

Obesity usually associated with some co-morbidities as diabetes, cancer, osteoarthritis, cardiovascular, respiratory and psychological disorders that adversely affect their quality of life (QOL)^26^, ^27^. However, obesity usually accompanied with poor mood and emotional well-being 28-30. Moreover, obese women usually have more deterioration in quality of life, low self-esteem and depression than obese men^31^. Weight reduction intervention is the most recent management policy for control of obesity via exercise, diet regimen and life style modification^32^.

The results of our study revealed that weight reducing program resulted in significant reduction in inflammatory parameters included IT-6, TNF-α and CRP, these findings agreed with Sandoval and Davis proved that bariatric surgery resulted in insulin sensitivity improved and reduced IL-6 correlated with weight loss^33^. In addition, Loria-Kohen et al reported that combined exercise and diet control resulted in reduced IT-6, TNF-α and CRP^34^. However, Balagopal et al. mentioned that three months of weight reducing program led to modulation of insulin resistance and IL-6^35^. Moreover, long-term exercise training resulted in weight loss and reduced TNF-α^36^. Similarly, weight reduction as result of exercise, diet control and liposuction led modulation of IT-6, TNF-α and CRP^37^,^38^. Reduction of visceral fat, pro-inflammatory monocytes numbers and increased regulatory T cells numbers are the probable anti-inflammatory causes of weight loss^39^-^42^.

Results of our study proved that weight loss improved asthma control test as reported in previous studies that proved that weight reduction was associated with better asthma control and improved lung function^43^-^53^. In addition, Pakhale et al stated that quality of life, lung function, airway hyperresponsiveness and asthma control improved following weight reduction program in obese asthmatic patients^54^. Moreover, Lv et al conducted a systematic review and concluded that weight loss was associated with improved asthma control through nonsurgical interventions^55^.

The principal finding in this study indicated that weight reducing program-improved subscales of QOL asthmatic patients. Many previous studies reported that weight loss improves HRQOL among obese subjects^53^-^58^, type 2 diabetes mellitus^59^ and osteoarthritis^56^. While, Ross et al. reported that a six months weight reducing program among 298 obese women that resulted in 9.4% weight loss resulted improvement in vitality scores and physical functioning^57^. However, Blissmer et al. proved that improvements in subscales of SF-36 was obtained in 144 overweight/obese adults after six months of weight reducing program that resulted in 5.6 kg of weight loss^58^. Moreover, Riesco and colleagues stated that modest loss of body weight following 16-week of aerobic exercise training improved QOL, physical and mental well-being in obese women^59^. In addition, several trials proved that a 6-month program resulted in about 10% weight reducing that associated with improved lung function and asthma control along with reduced hospital visit^60^-^62^.

The current study has important strengths and limitations. The major strength is the supervised nature of the study. Supervising food intake and physical activity removes the need to question compliance or to rely on food and activity questionnaires. Further, all exercise sessions were supervised and adherence to the diet and activities was essentially 100%. Moreover, the study was randomized; hence, we can extrapolate adherence to the general population. In the other hand, the major limitations are the small sample size in both groups may limit the possibility of generalization of the findings in the present study. Finally, within the limit of this study, Inflammatory cytokines and quality of life parameters improved with life style modification among obese asthmatic patients. Further researches are needed to explore the impact of weight reduction on quality of life and other biochemical parameters among obese asthmatic patients.

## Conclusion

Inflammatory cytokines and quality of life parameters improved with life style modification among obese asthmatic patients.

## Figures and Tables

**Table 2:** Mean value and significance of BMI, ACT, TNF-α, IL-6 and IL-10 of group (A)

Parameters	Mean ± SD		t-value	Significance
	Before	After		
**BMI** (kg/m^2^)	32.86 ± 4.62	28.12 ± 4.15*	6.42	P <0.05
**ACT (0–25)**	17 (16–21)	22 (18–25)*	6.46	P <0.05
**TNF-α** (pg/mL)	6.48 ± 1.91	4.13 ± 1.72*	5.47	P <0.05
**IL-6** (pg/mL)	3.16 ± 0.94	2.27 ± 0.85*	4.31	P <0.05
**IL-10** (pg/ml)	6.14 ± 1.75	8.23 ± 1.68*	5.52	P <0.05

BMI= Body mass index; ACT: Asthma control test; IL-6=Interleukin-6; IL-10= Interleukin-10 and TNF-α= Tumor necrotic factor-alpha; (*) indicates a significant difference between the two groups, P < 0.05.

(*) indicates a significant difference between the two groups; P < 0.05.

**Table 3:** Mean value and significance of SF-36 subscale scores in group (A) before and at the end of the study

	Mean ± SD		t-value	Significance
SF-36 subscale variables	Before	After		
**SF-36: Health transition**	2.86 ± 0.94	1.91 ± 0.83*	5.76	P<0.05
**SF-36: Physical functioning**	72.56 ± 7.32	80.31 ± 8.25*	7.14	P<0.05
**SF-36: Role functioning: Physical**	79.43 ± 7.91	86.42 ± 8.36*	6.93	P<0.05
**SF-36: Bodily pain**	74.25 ± 8.42	69.36 ± 7.23*	5.34	P<0.05
**SF-36: General health**	70.62 ± 7.94	76.65 ± 8.12*	7.21	P<0.05
**SF-36: Vitality**	55.23 ± 6.18	64.31 ± 7.26*	6.89	P<0.05
**SF-36: Social functioning**	87.16 ± 7.29	93.54 ± 8.15*	6.47	P<0.05
**SF-36: Role functioning: Emotional**	91.48 ± 9.11	85.63 ± 8.27*	5.26	P<0.05
**SF-36: Mental health**	83.17 ± 6.93	79.52 ± 6.41*	5.14	P<0.05
